# Echocardiographic Determination of Umbilical Catheter Tip Location Mitigates Complications: A Randomized, Controlled Trial

**DOI:** 10.3390/children12111509

**Published:** 2025-11-07

**Authors:** Yi-Jhen Lin, Yu-Chen Liu, Hsin-Chun Huang, Yao-Sheng Wang, Hwa-Shiu Wu, Yu-Han Su, Yu-Chen Hsu, I-Lun Chen

**Affiliations:** 1 Department of Pediatrics, Kaohsiung Chang Gung Memorial Hospital, College of Medicine, Chang Gung University, Kaohsiung City 833401, Taiwan; u102001463@cgmh.org.tw (Y.-J.L.); hhuang@cgmh.org.tw (H.-C.H.); s10101010@cgmh.org.tw (H.-S.W.); f810724@cgmh.org.tw (Y.-H.S.); yuchen16k@cgmh.org.tw (Y.-C.H.); 2 Department of Nursing, Kaohsiung Chang Gung Memorial Hospital, College of Medicine, Chang Gung University, Kaohsiung City 833401, Taiwan; yugen28@cgmh.org.tw; 3 School of Traditional Chinese Medicine, College of Medicine, Chang Gung University, Taoyuan 333323, Taiwan; 4 Department of Pediatrics, Chia-Yi Chang Gung Memorial Hospital, Chia-Yi 613016, Taiwan; kvnobulu2@cgmh.org.tw

**Keywords:** umbilical catheter, NICU, PoCUS, tip location

## Abstract

**Highlights:**

**What are the main findings?**

- Umbilical catheter position has been correlated with the incidence of catheter-related infection.
- PoCUS can identify the location of the umbilical catheter more accurately and imme-diately than traditional radiography.

**What is the implication of the main finding?**

- PoCUS should be considered as a standard tool for umbilical catheter placement.
- The use of PoCUS for catheter localization should be promoted in all NICUs.

**Abstract:**

**Background/Objectives**: Umbilical venous catheters (UVCs) and umbilical artery catheters (UACs) are essential for neonatal care, facilitating medication delivery, nutritional support, and blood pressure monitoring. However, malposition and prolonged catheter dwell time can lead to severe complications, including central line-associated bloodstream infections (CLABSIs). This study aims to evaluate the benefits of ultrasound in confirming catheter tip location, which may impact infection risk, and to assess the effectiveness of modification of the securing method. **Methods**: This prospective randomized controlled study was conducted from May 2022 to December 2024 at an NICU in Taiwan. Neonates requiring umbilical catheters were randomly assigned to three groups. In Group 1, the catheter length was calculated using a formula, X-ray confirmation was used, and the catheter was secured with traditional tape. In Group 2, ultrasound confirmation was used and the catheter was secured with FoamLite™ sterile dressing and transparent film. In Group 3, ultrasound confirmation was used and the catheter was secured with traditional tape. The outcomes were the rate of complications of the catheters. **Results**: Groups 2 and 3 demonstrated significantly lower malposition rates, microbial colonization, and CLABSI incidence compared to Group 1 (*p* = 0.001, 0.006, and 0.026, respectively). No significant difference was observed between Groups 2 and 3, suggesting that accurate tip positioning was more influential in reducing CLABSIs than the securing method itself. **Conclusions**: Ultrasound guidance improves catheter placement accuracy, minimizes malposition, lowers CLABSI risk, and reduces radiation exposure, supporting its broader implementation in NICUs.

## 1. Introduction

Umbilical catheterization is a common central line procedure used in neonates, particularly during the first few days of life. An umbilical venous catheter (UVC) can be rapidly inserted for medication administration during resuscitation or to provide central access after stabilization for parenteral nutrition, blood transfusion, and high-osmolality fluid administration. An umbilical artery catheter (UAC) is primarily used for blood pressure monitoring and blood sampling. These catheters can be lifesaving for critically ill neonates, but they also carry a risk of significant complications, including pericardial tamponade, limb ischemia, central line-associated bloodstream infections (CLABSIs), liver abscesses, and thrombi [1,2].

Our NICU has been performing approximately 70 umbilical catheter insertions each year for more than 20 years. Our catheter placement and maintenance skills are very proficient. However, complications from catheters still happen occasionally. Most complications are associated with malpositioned catheters and prolonged dwell time [1]. Catheter placement is a blind procedure, with insertion depth estimated using an equation based on body weight [3]. The catheter tip location is typically confirmed by a chest and abdominal X-ray after placement. However, X-ray imaging only provides a two-dimensional view, which may limit the accuracy in determining the catheter tip’s true position. Additionally, frequent X-ray examinations increase radiation exposure.

Ultrasonography offers real-time imaging and has been reported to have higher sensitivity and specificity than X-ray for detecting umbilical catheter tip location [4]. Moreover, ultrasound can be performed daily to monitor catheter displacement [5]. While ultrasonography requires practice, point-of-care ultrasound (PoCUS) for umbilical catheter evaluation is relatively straightforward and may not require extensive training [6].

CLABSIs are among the most significant complications associated with central catheters as it can lead to late-onset sepsis and prolonged hospitalization and has a negative influence on developmental outcomes in infants. Compared to peripherally inserted central catheters (PICCs), which are commonly used in NICUs [7], umbilical catheters have a significantly higher risk of CLABSIs [8] despite being placed using sterile techniques. The use of UVCs has been associated with colonization in 22–59% of cases and with CRBSIs in 3–20% of cases [9]. Currently, in most NICUs, umbilical catheters are secured with a tape bridge on the abdominal wall and are not always covered with sterile dressings, which could increase the risk of extraluminal colonization. While some umbilical catheter holders are available, they are often expensive and not widely accessible in many regions.

FoamLite™ is a commonly used sterile medical material specifically designed for chronic wound care, such as pressure sores. It is a sterile polyurethane foam with high absorptive capacity for secretions, thereby reducing local moisture and limiting bacterial proliferation. Moreover, it is cost-effective and accessible. It could potentially be used to cover the umbilical stump and securely maintain catheter sterility.

This randomized controlled study aims to evaluate the benefits of ultrasound in confirming catheter tip location, which could reduce complications of catheters, and to assess the effectiveness of FoamLite™ dressing in preventing CLABSIs in umbilical catheters used in NICUs.

## 2. Materials and Methods

This prospective randomized controlled study was conducted from May 2022 to December 2024 and was approved by the Institutional Review Board of the Chang Gung Medical Foundation in Taoyuan, Taiwan, on 12 May 2022. The approval number was 202200365A3. Neonates were considered eligible for the study if they required UVC and UAC insertion as part of standard clinical care upon admission, contingent upon obtaining written informed consent from their parents. Neonates with significant umbilical cord anomalies, hydrops, or those who experienced unsuccessful umbilical catheter placements were excluded from the study. Finally, the enrolled participants were randomly assigned to groups using computer-generated random numbers.

The size of the umbilical artery or vein catheter was selected based on the infant’s body weight, using 2.5-French, 3.5-French, or 5-French catheters. In this study, we utilized Argyle™ polyurethane umbilical vessel catheters (Cardinal Health, Inc., Dublin, OH, USA). Catheter placements were performed by a dedicated team consisting of pediatricians with at least two years of clinical experience and senior pediatric residents. The anticipated catheter insertion length was calculated using a standardized equation [3].

Sonography was performed by the same senior pediatric resident, who had at least two months of experience performing PoCUS. This resident evaluated the catheter tip position and made real-time adjustments under ultrasound guidance when necessary. The entire procedure was performed under sterile conditions. The clinicians put on sterile gloves, gowns, masks, and caps before the procedure. The umbilical cord and peri-umbilical area was sterilized with povidone–iodine and the baby was covered with a sterile drape.

**Figure 1:**
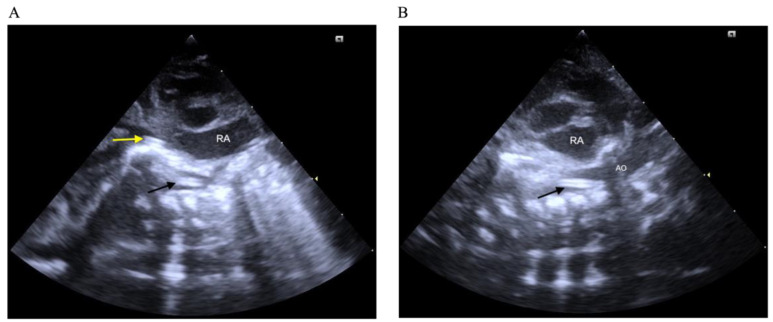
The proper positioning of the UAC and UVC: (**A**) the yellow arrow indicates that the UVC is at the junction of the inferior vena cava and right atrium and the black arrow shows that the UAC is within the thoracic aorta above the diaphragm (**A**,**B**). RA: right atrium; AO: aorta.

Until June 2024, a Philips ultrasound system with a sector probe (frequency range: 5–12 Hz) was used; thereafter, a Siemens Acuson system (Germany) with a sector probe (frequency range: 2.7–8 Hz) was implemented. The tip of the UVC was identified using the subxiphoid right parasagittal view. The US probe was placed longitudinally at the right subcostal area to visualize the catheter’s tip at the inferior vena cava to right atrial junction. The subxiphoid longitudinal views were used for visualizing the UAC tip (Figure 1). The proper position of catheters in US was defined as being in the thoracic aorta, 0.5 to 1 cm above the diaphragm for UACs and at the inferior vena cava to right atrial junction, outside of the liver, for UVCs. A small volume of saline (approximately 1 mL) was injected to confirm the catheter tip location. Malposition was defined as any deviation from these criteria, including UVC tips located within the liver, hepatic vein, or portal vein, and UAC tips positioned outside the thoracic aorta between the sixth and ninth thoracic vertebrae or too low in the abdominal aorta. Although low-lying placement of UACs and UVCs is also feasible, our NICU consistently adopts the high position approach.

**Figure 2:**
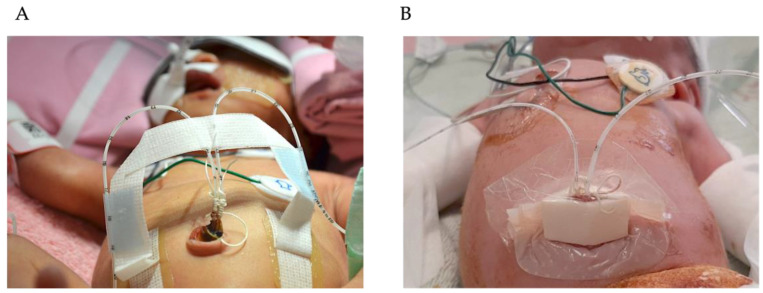
Methods for securing umbilical catheters: (**A**) catheters secured utilizing traditional tape bridging; (**B**) catheters secured with FoamLite™ dressings combined with a transparent film.

Utilizing a mobile digital X-ray imaging station, we conducted an anteroposterior X-ray of the neonate’s chest and abdomen following catheter insertion to ascertain the catheter tip location. This X-ray served as the definitive standard for assessing catheter tip placement in the three groups. Radiographic images were interpreted by a neonatologist uninvolved in the catheter placement. The positioning of the catheter tip on the X-ray above the right diaphragm near the right atrium was considered correct for the UVC [10], and positioning between the sixth and ninth thoracic vertebrae was correct for the high position of the UAC [11]. Prior to adjustment of the catheters, the withdrawn length of the catheter was measured on the X-ray film. Following sterilization of the umbilical cord, the catheter was unable to be advanced and could only be withdrawn. The duration of ultrasound and radiographic examinations was recorded from the initiation of the PoCUS exam (when the probe was positioned on the chest wall and real-time imaging was displayed on the ultrasound monitor) to the end of the PoCUS exam (upon confirmation of the proper tip location). This was followed by ordering and completion of the X-ray exam, as outlined in our previous study [12,13]. All enrolled neonates were randomly assigned to one of three groups:

Group 1: Catheter insertion length was calculated using a standardized formula, and catheters were secured with traditional tape bridging (Figure 2A), with tip location confirmed by radiography post-placement.

Group 2: Sonography was used for tip location evaluation, and catheters were secured using FoamLite™ dressings combined with a transparent film (Figure 2B).

Group 3: Sonography was used for tip location evaluation, and catheters were secured using traditional tape bridging (Figure 2A).

Catheter colonization was defined as the growth of ≥15 colony-forming units of any single microorganism on semi-quantitative culture of the catheter (UAC or UVC) tip without laboratory-confirmed blood stream infection of the patient [9]. Catheter tip cultures were performed using the roll-plate (roll-out) semi-quantitative culture method [14]. A CLABSI is defined by the Centers for Disease Control and Prevention as the recovery of a pathogen from a blood culture in a patient who had a central line at the time of infection or within 48 h prior to infection onset [15]. Umbilical catheters (UAC and UVC) were always placed at the same time and similar sterilization procedures were performed but the catheters were sometimes removed separately. Thus, the indwelling duration of catheters was defined as the time from the date of placement to the last catheter withdrawal.

Gestational age, preterm delivery, birth weight, sex, Apgar score, catheter dwelling duration, procedural time, malposition, catheter tip culture, and blood culture results were analyzed across the three groups using one-way ANOVA with Bonferroni correction for continuous variables and Chi-squared tests for categorical variables. Additionally, multinomial logistic regression was conducted to evaluate the influence of catheter configuration and procedural approach on umbilical catheter outcomes. All statistical analyses were performed using IBM SPSS Statistics version 26 (IBM Corp., Armonk, NY, USA).

## 3. Results

**Figure 3:**
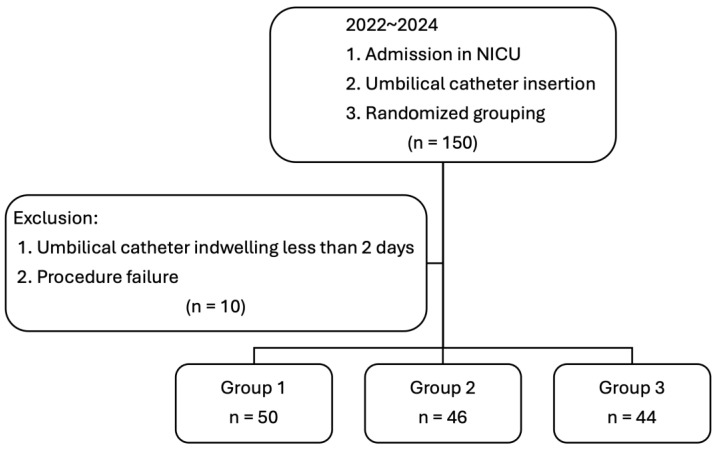
The study flow chart. The randomized controlled study consisted of three groups. Group 1 utilized traditional tape bridging to secure the catheter, with tip location verified through post-placement radiography. Group 2 employed FoamLite™ dressings combined with a transparent film to secure the catheter, with tip positioning confirmed by sonography. Group 3 also used traditional tape bridging to secure the catheter, with tip location evaluated via sonography.

A total of 150 neonates were included in this study and randomly assigned to three groups, with 50 neonates in each group. Eight neonates had umbilical catheters in place for less than two days, and catheter insertion failed in two cases. Ultimately, 140 neonates were included in the final analysis: 50 in Group 1, 46 in Group 2, and 44 in Group 3 (Figure 3). Gestational age, the number of preterm neonates, birth weight, sex, Apgar scores, neonatal antibiotics use, and indwelling time did not significantly differ among the three groups. However, the frequency of prenatal antibiotic treatment of the mother was significantly higher in Group 2 than Groups 1 and 3 (*p* = 0.001). Regarding the catheter parameters, the time required for the first confirmation of tip location, malposition rate, microorganism colonization, and CLABSIs were significantly higher in Group 1 compared to Groups 2 and 3 (*p* < 0.001, 0.001, 0.006, and 0.026, respectively) (Table 1). No significant differences were observed between Groups 2 and 3.

Further analysis using multinomial logistic regression for demographic data of infants and significant variables in Table 1 found that malposition rate and time to first tip confirmation showed the most significant differences when comparing Group 2 vs. Group 1 (*p* = 0.03, OR: 0.253, 95% CI: 0.073–0.874 and *p* < 0.001, OR: 0.970, 95% CI: 0.954–0.986, respectively) and Group 3 vs. Group 1 (*p* = 0.005, OR: 0.131, 95% CI: 0.032–0.533 and *p* < 0.001, OR: 0.923, 95% CI: 0.889–0.960, respectively) (Table 2). The frequency of antenatal antibiotic treatment of the mother was significantly lower in Group 3 than in Group 1 (*p* = 0.008, OR: 0.240, 95% CI: 0.084–0.688)

## 4. Discussion

This randomized controlled trial revealed that the conventional approach, which confirms tip location through radiography, required a longer confirmation period and was linked to higher rates of malposition, microorganism colonization, and CLABSIs when compared to sonography. Sonography offers real-time imaging, facilitating sterile adjustments to catheter insertion length.

When comparing the radiographic group (Group 1) to the sonographic groups (Groups 2 and 3), the latter demonstrated superior accuracy in tip location and a reduced rate of malposition. In Group 1, 50% of the catheters necessitated reopening of the securing area for tip adjustment following X-ray confirmation, which may have increased the risk of microorganism invasion. In Group 1, catheter malposition was identified in 25% (1/4) of bloodstream infection cases and 60% (6/15) of bacterial colonization cases. The main distinction between Group 2 and Group 3 was the securing method: FoamLite™ dressing versus tape bridging. The tape bridge utilized adhesive tape across the umbilicus to secure the catheter to the abdominal wall (Figure 2A), while the FoamLite™ dressing involved a sterile FoamLite™ covering on the umbilical stump (Figure 2B). Despite the sterility of the FoamLite™ dressing, it did not significantly reduce bacterial colonization or the incidence of CLABSIs. These results indicate that promptly and accurately confirming the tip location of an umbilical catheter is more effective in lowering the risk of CLABSIs than the choice of securing method.

The significantly lower rate of antenatal antibiotic use in Group 3 implies that maternal bacterial infections were less prevalent in this group, which may have contributed to the observed differences in neonatal outcomes. However, there were no significant differences in the incidence of maternal fever, chorioamnionitis, or premature rupture of membranes among the three groups. Therefore, the potential impact of maternal bacterial infections on neonatal bloodstream infections was likely minimal.

Sonography is recommended for more accurate catheter placement, reducing the need for repeated imaging and catheter repositioning, which in turn decreases the risk of CLABSIs. Many researchers have described sonography as a tool with higher sensitivity, higher specificity, greater convenience, and lower cost compared to X-ray for detecting the umbilical catheter tip location. Rossi et al. reported that the accuracy of UVC placement increased from 46.2% to 58.8% when ultrasound guidance was utilized in place of Shukla’s formula [16]. Xie et al. demonstrated that PoCUS can be used to effectively monitor catheter tip displacement [5], which frequently occurs within the first 2–4 days post-placement, potentially leading to misalignment and increased complication risks. Additionally, Ponin et al. found that the use of PoCUS increased correct catheter placement rates from 20.4% for umbilical venous catheters and 33.3% for umbilical arterial catheters to 64.0% and 83.8%, respectively [17].

A significant benefit of echocardiographic catheter confirmation noted in this study was the decrease in rates of CLABSIs. Conventional catheter placement methods carry a higher risk of bloodstream infections due to repeated adjustments and extended procedural times. Jansen et al. highlighted that CLABSIs continue to be a prevalent challenge in NICUs, showing a six-fold variation in incidence among different centers, which is influenced by both catheter dwell time and insertion techniques [8]. However, the use of a tape bridge to secure the catheter without sterile dressing coverage in this study did not significantly elevate CLABSI rates or catheter colonization. This practice aligns with World Health Organization recommendations, which advocate for keeping the umbilical cord dry, without antiseptic treatment, for healthy neonates born in settings with lower neonatal mortality rates [18]. A case–control study further illustrated that there was no significant difference in complication rates between dry cord care (keeping the umbilical cord dry with sterile gauze around the cord base on the first day to absorb any bloody secretions) and the application of 70% alcohol [19]. Although the umbilical catheter is inserted through the umbilical cord, the umbilical stump is cleaned with povidone–iodine before the procedure and during each instance of catheter care. Thus, there was no increase in catheter-related bloodstream infections, even in the absence of sterile dressing coverage.

During their NICU stay, patients receive between 0 and 159 X-ray exposures [20]. It was observed that patients with lower gestational ages and lower birth weights consistently had a higher number of X-ray examinations. While long-term complications related to radiation exposure in premature neonates have not been extensively documented, there have been reports of carcinogenesis, circulatory issues, and metabolic diseases in humans [21,22]. Notably, sonography does not involve radiation. Rubortone et al. emphasized that ultrasound can reduce the dependency on X-ray imaging [6], thereby minimizing radiation exposure—an essential consideration due to the increased sensitivity of neonates to ionizing radiation. Although some researchers have pointed out that echocardiographic confirmation of umbilical venous catheter placement necessitates specialized skills and a learning curve [23], PoCUS for catheter localization presents numerous advantages for neonates and should be widely implemented in NICUs.

This study has several limitations. The proficiency of clinicians in performing PoCUS varies. Targeted neonatal echocardiography for catheter tip localization requires structured competency assessments to ensure consistency in practice [24]. In this investigation, sonographic evaluations were carried out by a senior resident pediatrician with a minimum of two months of experience in PoCUS to minimize variability among operators. Additionally, another limitation arose when the ultrasound machine was upgraded to a new model towards the end of the study, which offered superior resolution and reduced the time needed for tip confirmation. Nonetheless, patients were randomly assigned to groups, thus minimizing any potential bias.

## 5. Conclusions

PoCUS can accurately identify the location of the catheter tip, which can markedly decrease the risks of malposition and delayed verification, and is associated with a decrease in infection compared with conventional radiographic methods. Utilizing PoCUS for standard umbilical catheters placement should be considered in all NICUs.

## Figures and Tables

**Table 1:** Demographic data of the perinatal period and complications of catheters.

Variable	Group 1 (n = 50)	Group 2 (n = 46)	Group 3 (n = 44)	*p* Value
Birth weight (g)	1675.3 ± 923.07	1685.0 ± 824.8	1788.6 ± 903.4	0.795
Sex (F/M)	28/22	22/24	22/22	0.774
Gestational age (week)	31.44 ± 4.79	32.22 ± 4.38	32.91 ± 4.80	0.314
Preterm delivery (n, %)	41 (82)	36 (78.3)	31 (70.5)	0.495
Apgar score at 1 min	5.24 ± 2.64	5.52 ± 2.25	5.59 ± 2.39	0.749
Apgar score at 5 min	7.20 ± 2.24	7.41 ± 2.09	7.43 ± 2.16	0.837
Cesarean section delivery	30 (60)	35 (76)	29 (66)	0.208
Maternal fever	10 (20)	5 (10.9)	5 (11.4)	0.281
Premature rupture of membranes	10 (20)	7 (15.2)	12 (27.3)	0.347
Chorioamnionitis	11 (22)	5 (10.8)	12 (27.2)	0.132
Antenatal steroid	26 (52)	23 (50)	23 (52.3)	0.809
Antenatal magnesium	32 (64)	24 (52.1)	19 (43.2)	0.222
Antenatal antibiotics	42 (84)	42 (91.3)	24 (54.5)	0.001
Both UAC and UVC insertion	21 (42.0)	27 (58.7)	27 (61.4)	0.167
Duration of indwelling umbilical catheters (days)	4.76 ± 3.17	4.72 ± 2.12	4.25 ± 1.93	0.557
Duration of confirmed tip location (minutes)	102.12 ± 116.9	23.72 ± 39.4	11.63 ± 9.82	<0.001
Malposition (n, %)	25 (50)	10 (21.7)	8 (18.2)	0.001
Neonatal antibiotic use	50 (100)	46 (100)	44 (100)	-
Catheter colonization (n, %)	15 (30)	5 (10.9)	3 (6.8)	0.006
Catheter related bloodstream infection (n, %)	4 (8)	0	0	0.026

The data are presented as mean ± standard deviation and number (percentage). One-way ANOVA was used for continuous variables and Chi-squared tests for categorical variables. F/M: female/male; UAC: umbilical artery catheter; UVC: umbilical vein catheter.

**Table 2:** Multinomial logistic regression analysis comparing the outcomes of Groups 2 or 3 against Group 1.

Multiple Regression Model				
	Group 2 vs. Group 1		Group 3 vs. Group 1	
	*p* Value	Odds Ratio (95% CI)	*p* Value	Odds Ratio (95% CI)
Birth weight	0.078	0.998 (0.996–1.000)	0.147	0.999 (0.997–1.001)
Sex (F/M)	0.479	1.510 (0.482–4.732)	0.613	1.399 (0.381–5.144)
Gestational age	0.144	1.265 (0.923–1.733)	0.166	1.270 (0.906–1.779)
Preterm delivery	0.195	0.202 (0.018–2.267)	0.264	0.239 (0.019–2.939)
Apgar score at 1 min	0.937	1.020 (0.625–1.666)	0.591	1.162 (0.671–2.012)
Apgar score at 5 min	0.821	1.065 (0.616–1.843)	0.919	0.969 (0.530–1.771)
Antenatal antibiotics	0.360	1.955 (0.466–8.204)	0.008	0.240 (0.084–0.688)
Duration of indwelling umbilical catheters	0.292	1.177 (0.869–1.596)	0.280	1.212 (0.855–1.720)
Duration of confirmed tip location	<0.001	0.970 (0.954–0.986)	<0.001	0.923 (0.889–0.960)
Malposition	0.030	0.253 (0.073–0.874)	0.005	0.131 (0.032–0.533)
Catheter colonization	0.578	0.651 (0.143–2.955)	0.347	0.404 (0.061–2.675)

F/M: female/male. CI: Confidence interval.

## Data Availability

The data presented in this study are available on request from the corresponding author. The data are not publicly available due to ethical considerations.

## Abbreviations

The following abbreviations are used in this manuscript:

UVCs	Umbilical venous catheters
UACs	Umbilical artery catheters
CLABSI	Central line-associated bloodstream infection
PoCUS	Point-of-care ultrasound
PICCs	Peripherally inserted central catheters
